# Serum butyrylcholinesterase in type 2 diabetes mellitus: a biochemical and bioinformatics approach

**DOI:** 10.1186/1476-511X-4-18

**Published:** 2005-09-08

**Authors:** GR Sridhar, G Nirmala, Allam Apparao, AS Madhavi, S Sreelatha, J Sudha Rani, P Vijayalakshmi

**Affiliations:** 1 Department of Computer Science and Systems Engineering, Andhra University, Visakhapatnam, India; 2 Endocrine and Diabetes Centre, 15-12-16 Krishnanagar, Visakhapatnam 530 002, India

**Keywords:** metabolic syndrome, type 2 diabetes, dyslipidemia, phylogenetic tree, evolutionary distance

## Abstract

**Background:**

Butyrylcholinesterase is an enzyme that may serve as a marker of metabolic syndrome. We (a) measured its level in persons with diabetes mellitus, (b) constructed a family tree of the enzyme using nucleotide sequences downloaded from NCBI. Butyrylcholinesterase was estimated colorimetrically using a commercially available kit (*Randox Lab, UK*). Phylogenetic trees were constructed by distance method (*Fitch and Margoliash *method) and by *maximum parsimony *method.

**Results:**

There was a negative correlation between serum total cholesterol and butyrylcholinesterase (-0.407; p < 0.05) and between serum LDL cholesterol and butyrylcholinesterase (-0.435; p < 0.05). There was no statistically significant correlation among the other biochemical parameters. In the evolutionary tree construction both methods gave similar trees, except for an inversion in the position of *Sus scrofa *(M62778) and *Oryctolagus cuniculus *(M62779) between Fitch and Margoliash, and maximum parsimony methods.

**Conclusion:**

The level of butyrylcholinesterase enzyme was inversely related to serum cholesterol; dendrogram showed that the structures from evolutionarily close species were placed near each other.

## 1. Introduction

The enzyme butyrylcholinesterase (BChE; EC 3.1.1.1.8) has a well-defined pharmacologic function in hydrolyzing succinylcholine, a muscle relaxant used in anesthetic practice. It could have other roles, though much less well defined, such as modulating the phenotypic expression of dyslipidemia and metabolic syndrome. Serum levels of the enzyme are affected by dietary fat, obesity, hyperlipidemia and diabetes mellitus [1].

With genomic sequences from many species being available in the public domain [2], it is possible to annotate proteins in evolutionarily terms. Such analysis is particularly useful for proteins with poorly defined physiological role, such as BchE. A phylogenetic analysis of amino acid/ nucleotide sequences could throw light on 'how the family might have been derived during evolution' [3]. The analysis is represented as an evolutionary tree, which is a two-dimensional graph; it shows evolutionary relationships of genes from different organisms.

Here we (a) studied the level of BChE among persons with diabetes mellitus (b) constructed the phylogenetic tree of 25 BChE genes from publicly available sequence data.

## 2. Materials and methods

We studied 30 individuals with clinical type 2 diabetes mellitus at our Centre in southern India (14 men, 16 women; age 51.9 years+/- 7.9 years, duration of diabetes 6.6 years+/-3.74 years). Butyrylcholinesterase was estimated colorimetrically using a commercially available kit (*Randox Lab, UK*). Serum insulin and C-peptide were measured by radioimmunoassay, and lipids (total cholesterol, triglycerides, HDL cholesterol) by colorimetry.

Results are expressed as mean+/- SD. Multiple correlation analysis was done by SPSS package (v10.5). A p value of < 0.05 was taken as significant.

Twenty five sequences of BchE gene were retrieved from National Centre for Biotechnology Information (NCBI): *Rattus norvegicus *(Accession no: NM_022942), *Mus musculus *(NM_009738), *homo sapiens *(NM_000055), *homo sapiens *(BC018141), *homo sapiens *(BC008396), *Sus scrofa *(AF222914), *Gallus gallus *(AJ306928), *Panthera tigris tigris *(AF053484), *Felis catus *(AF053483), *Equus caballus *(AF178685), *Rattus norvegicus *(AF244349), *Oryctolagus cuniculus *(X52092), *Oryctolagus cuniculus *(X52091), *Oryctolagus cuniculus*(X52090), *homo sapiens *(M16541), *Oryctolagus cuniculus*(U04814), *homo sapiens *(M16474), *Canis Familiaris *(M62411), *Bos taurus *(M62410), *Macaca mulatta *(M62777), *Ovis aries *(M62780), *Oryctolagus cuniculus *(M62779), *Sus scrofa *(M62778), *Mus musculus *(NM_009599), and *homo sapiens *(NM_000446).

Phylogenetic trees were constructed using two methods: distance method (*Fitch and Margoliash *method) and *maximum parsimony *method (MP) [3].

In the distance method (*Fitch and Margoliash*) the sequences were aligned by local pair-wise method and a distance score obtained for each pair of sequences (25 × 25 matrix). The scores were represented as a distance matrix; the most closely related sequences in the matrix were identified and represented as a tree/branch. The average distance between these sequences and each of the other sequences was calculated to obtain a new distance matrix. This process was repeated till all sequences were added to the tree.

In the maximum parsimony method sequences were aligned by global pairwise method, possible trees constructed, parsimony cost of each tree calculated and the one with minimum cost identified as the optimal tree, which was the selected output.

## 3. Results

The results are presented in Table 1. There was a negative correlation between serum total cholesterol and butyrylcholinesterase (-0.407; p < 0.05) and between serum LDL cholesterol and butyrylcholinesterase (-0.435; p < 0.05). There was no statistically significant correlation among the other biochemical parameters.

**Table 1:** Biochemical parameters in Type 2 diabetes subjects

Parameter	Value
Glycosylated hemoglobin (n: 30)	9.23+/-0.88%,
Total cholesterol (n: 30)	197.7+/-45.06 mg/dl,
Triglycerides (n: 30)	166.6+/-76.88 mg/dl,
LDL cholesterol (n: 30)	129.56+/-45.39 mg/dl,
HDL cholesterol (n: 30)	35.3+/-6.83 mg/dl,
Serum insulin (n: 29)	13.96+/-12.13 uiu/ml
C-peptide (n: 28)	0.21+/-0.11 ng/ml.
Serum BChE level (n: 29)	3401.72+/-924.125 U/L

**Figure 1:**
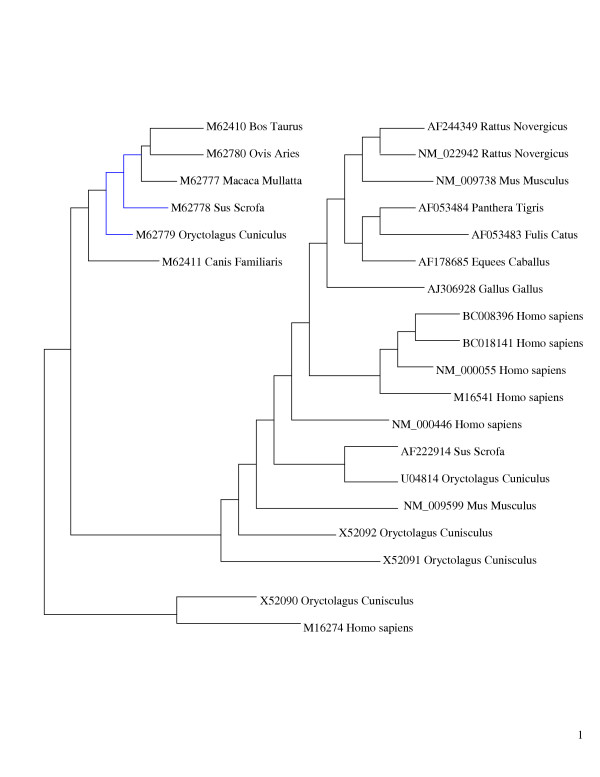
Phylogenetic tree constructed using Fitch-Margoliash for bche and variants (n:25).

**Figure 2:**
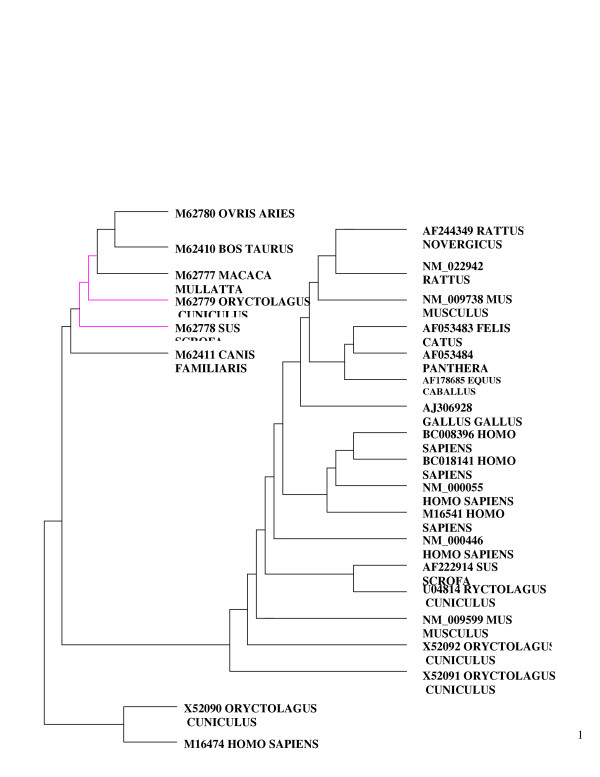
Phylogenetic tree: Maximum parsimony for bche and variants (n:25).

Both methods of tree construction gave similar dendrograms, except for an inversion in the position of *Sus scrofa *(M62778) and *Oryctolagus cuniculus *(M62779) between Fitch and Margoliash, and maximum parsimony methods (Figs 1, 2). There were two main subdivisions in the phylogenetic tree, with homo sapiens proteins together, and a pairing together of *rattus *(A5244349, NM_022942), *Felicus catus *(AF053483), *Panthera tigris *(AF053485), and *Oryctolagus cunniculus *(X52092, X53091)

## 4. Discussion

BChE breaks down ester type of neuromuscular blocking agents used in anesthesia [4]. It may also play a role in lipid metabolism [5], and may be affected by dietary fat [6]. Similarly, diabetes mellitus has been shown to alter the levels of the enzyme. BchE, which can affect cell membrane oxidative stress and fluidity [1], is structurally homologous to lipase [7]. The ratio of BChE to HDL cholesterol could be a marker of cardiovascular risk in the metabolic syndrome [8]. However recent studies have suggested BchE may not have a direct pathophysiological role in the development of metabolic syndrome [9], but may be considered as secondary markers for this syndrome in obese individuals with the CHE2 C5- phenotype [10]. In consonance with earlier studies, level of butyrylcholinesterase was related to changes in serum lipids; however there was a relationship to serum triglycerides in a previous study [9] and to serum cholesterol in ours.

BChE activity could also be used to select the drug for treatment of hypertriglyceridaemia in type 2 diabetes mellitus [11]. Similarly diabetes was one of the risk factors for coronary artery disease that independently correlated with BchE activity [12].

In the construction of evolutionary trees, the differences in *Sus scrofa *and *Oryctolagus cuniculus *was the only variant in the two methods. The sequences being similar, such an output is to be expected. Consistent with an earlier report [13], the genes of the following were close to each other: cow (*Bos taurus *M62410) and sheep (*Ovis aries *M62780), pig (*Sus scrofa *M62778) and dog (*Canis familiaris *M62411), rabbit (Oryctolagus cuniculus U04814) and house mouse (*Mus musculus *NM_009599).

Animal BchE gene structure was shown to be similar to the human gene [13]. The rate of evolution was reported to be rapid, but not more than other proteins with well-known function. BchE evolution had a 2.2 unit evolutionary period (ie, 2.2 million years for a 1% amino acid change) [13].

A broad based phylogenetic synthesis using combined and separate analysis of published molecular and morphological source phylogenies showed generally comparable tree structures [14]: tiger, cat and horse together.

Despite the limitations in the study, it establishes proof of concept that clinical and biochemical parameters can be linked to data from nucleotide sequences; an evolutionary perspective may be obtained, especially for proteins with poorly defined physiological functions.

In conclusion we (a) found a negative correlation between serum total cholesterol and butyrylcholinesterase and between serum LDL cholesterol in persons with type 2 diabetes (b) generated a phylogenetic tree from 25 sequences of BchE using distance method (*Fitch and Margoliash *method) and *maximum parsimony *method.
